# Impact of bromodomain‐containing protein 4 (BRD4) and intestine‐specific homeobox (ISX) expression on the prognosis of patients with hepatocellular carcinoma' for better clarity

**DOI:** 10.1002/cam4.4094

**Published:** 2021-06-25

**Authors:** Kai‐Ting Chuang, Shen‐Nien Wang, Shih‐Hsien Hsu, Li‐Ting Wang

**Affiliations:** ^1^ Graduate Institute of Medicine College of Medicine Kaohsiung Medical University Kaohsiung Taiwan; ^2^ Division of General and Digestive Surgery Department of Surgery Kaohsiung Medical University Hospital Kaohsiung Taiwan; ^3^ Department of Surgery College of Medicine Kaohsiung Medical University Hospital Kaohsiung Taiwan; ^4^ School of Medicine, College of Medicine Kaohsiung Medical University Kaohsiung Taiwan; ^5^ Department of Medical Research Kaohsiung Medical University Hospital Kaohsiung Medical University Kaohsiung Taiwan; ^6^ Department of Life Science National Taiwan Normal University Taipei Taiwan; ^7^ Center of Applied Genomics Kaohsiung Medical University Kaohsiung Taiwan

**Keywords:** BRD4, HCC, ISX

## Abstract

Epigenetic regulation is important for cancer tumor metastasis and progression, including lung and liver cancer. However, the mechanism of epigenetic regulation in liver cancer leaves much to be discussed. According to a previous study, p300/CBP‐associated factor (PCAF) mediated epithelial–mesenchymal transition (EMT) and promotes cancer metastasis by recruiting intestine‐specific homeobox (ISX) and bromodomain‐containing protein 4 (BRD4) in lung cancer. To figure out whether the three genes are also expressed in patients with hepatocellular carcinoma (HCC) or not, and their correlation with patients’ outcome, BRD4, PCAF, and ISX messenger RNA (mRNA) expression levels in 377 patients with HCC were investigated using quantitative polymerase chain reaction and confocal fluorescence imaging. The correlation of the gene expression (PCAF, ISX, and BRD4) in liver cancer is also being investigated. Here, we show that the mRNA expression of PCAF, BRD4, and ISX in 377 paired specimens from patients with HCC, and the adjacent normal tissues exhibited a tumor‐specific expression pattern, highly correlated with disease pathogenesis, patient survival time, progression stage, and poor prognosis. The results show that ISX and BRD4 can potentially be a target for improving the survival rate.

## Graphical abstract

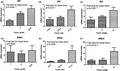

## 1 INTRODUCTION

Hepatocellular carcinoma (HCC), the fifth most commonly occurring cancer and the third leading cause of cancer‐related deaths every year worldwide.^1^, ^2^ In Taiwan, it is the second leading cause of preventable deaths after lung cancer.^3^ From the clinical medicine perspective, reduction in the mortality rate of liver cancer and improvement of the quality of life of liver cancer patients, the detection and treatment of liver cancer are all issues that need to be addressed. The most common type of liver cancer is HCC, which occurs most often in people with chronic liver diseases, for example, cirrhosis caused by hepatitis B or hepatitis C infection.^4^

One third of cirrhotic patients is estimated to develop liver cancer during their lifetime,^5^ with a 1–8% annual incidence reported in long‐term follow‐up studies (e.g., 2% in HBV‐infected cirrhotic patients and 3–8% in HCV‐infected cirrhotic patients).^6^ Cancer is a genetic disease and an epigenetic disease, and people take the epigenetic aspect being used as a target for cancer treatment is gaining more attention.^7^, ^8^ Epigenetic regulation is defined as the expression of genetic changes through the modification of chromatin structure without changing the basic nucleotide sequence.^9^, ^10^ For example, in recent research, overall cell metabolism can be regulated through acetylation is reported. ^11^, ^12^, ^13^ The epigenetic regulation in cancer is discussed in several papers. Intestinal‐specific homeobox (ISX) is proved to mediate a feed‐forward loop integrating tryptophan catabolism, inflammation, and also immune suppression in HCC.^14^, ^15^ ISX is a proto‐oncogene and known to promote the proliferation, tumorigenesis, and immune tolerance of HCC via proinflammatory cytokine‐mediated upregulation of cyclin D1 and E2F1.^16^ In many cancer such as liver cancer, lung cancer, and prostate cancer, bromodomain‐containing protein 4 (BRD4) is a transcriptional and epigenetic regulator t playing a critical role during carcinogenesis and embryogenesis.^17^, ^18^, ^19^, ^20^ In lung cancer, p300/CBP‐related factor (PCAF) acetylates the ISX–BRD4 complex, unpacks chromatin, and activates the expression of EMT regulators through acetylation of histone H3, eventually promoting EMT and metastasis. Bromodomain‐containing protein 4 (BRD4) acts as a chromatin reader and mediates the binding of acetylated histones. It helps to form a multiprotein complex to connect the super‐enhancer and promoter. p300/CBP‐associated factor (PCAF) is a member of the GCN5‐related protein acetyltransferase *N*‐acetyltransferase family with histone acetyltransferase activity, which exhibits ambiguous or controversial functions in tumorigenesis^18^, ^21^ However, whether the PCAF–ISX–BRD4 mechanism is restricted to non‐small‐cell lung cancer (NSCLC) or it is also active in other tumor entities during malignant transformation remains unclear.

To explore whether or not ISX, BRD4, and PCAF are also co‐expressed in HCC and modulate the outcome of tumor severity, in this study, we collected the hepatitis specimens from 377 patients with HCC to explore how PCAF, BRD4, and ISX, respectively, and collaboratively impact on liver cancer tumors and the correlation with the prognosis of patients with HCC are discussed. Here we mainly use quantitative polymerase chain reaction (qPCR) technology and some cell biology experiments such as western blot to show the co‐expression and the correlation between the expression of the three genes in HCC and their impact on the prognosis of liver cancer.

## 2 RESULT

### 2.1 Significant differences in cancer stages, grade, and tumor sizes were observed between patients with high and low levels of ISX or BRD4 expression

Patient characteristics are shown in Table 1. The number of participants enrolled in this study was 377 (288 males and 89 females). The mean age of patients with HCC was 61.2 years.

**TABLE 1:** Basic Characteristic of 377 HCC patients according to mRNA expression of ISX, BRD4, and PCAF

	Total	*ISX*		*BRD4*		*PCAF*	
		Low	High	Low	High	Low	High
N=	377	286 (%)	91 (%)	305 (%)	72 (%)	275 (%)	102 (%)
Age (Mean ± SD)							
	61.2 ± 11.3	60.9 ± 11.7	62.4 ± 9.7	61.1 ± 11.3	61.9 ± 11.3	60.8 ± 11.9	62.6 ± 8.9
Sex							
Male	288	215 (75.5)	73 (78.9)	231 (76.2)	57 (77.0)	204(74.7)	84 (80.8)
Female	89	70 (24.5)	19 (21.1)	72 (23.8)	17 (23.0)	69 (25.3)	20 (19.2)

Patients with HCC were classified into “low” and “high” groups according to survival receiver operator characteristic (ROC) curve analysis. The threshold values of ISX, BRD4, and PCAF separately were 2.0, 3.0, and 2.2 times the mRNA expression in HCC than that of the neighboring healthy tissues. The high and low ISX group contained 286 and 91 patients, respectively, and the low BRD4 group contained 305 patients. Meanwhile, the high and low PCAF groups contained 102 and 275 patients, respectively.

To explore the clinical impact of PCAF, ISX, and BRD4 signals in HCC, 377 paired HCC samples (tumors along with neighboring healthy liver tissues) were obtained and analyzed.

In Table 2, significant differences in cancer stages, grades, and tumor sizes were observed between patients with high and low levels of ISX or BRD4 expression.

**TABLE 2:** Clinical and pathological characteristics of 377 HCC patients according to mRNA expression of ISX and BRD4

	*Total*	*ISX*			*BRD4*		
		Low	High	*p*	Low	High	*p*
N=	377	286 (%)	91 (%)		305 (%)	72 (%)	
Stage							
I	207	165 (57.6)	42 (47.3)	0.0484*	175 (58)	32 (41.9)	<0.001*
II	107	81 (28.3)	26 (28)		91 (29.7)	16 (23)	
III	63	40 (14.1)	23 (24.7)		39 (12.3)	24 (35.1)	
Grade							
I	49	41 (14.5)	8 (7.8)	0.0268*	39 (13)	10 (12.5)	0.9073
II	247	194 (67)	53 (60)		201 (65.3)	46 (65.3)	
III	79	50 (18)	29(31.1)		63 (21)	16 (22.2)	
IV	2	1 (0.3)	1 (1.1)		2 (0.7)	0 (0)	
Size (cm)							
<2	51	43 (15)	8 (8.8)	0.0228*	45 (14.7)	6 (8)	0.0001*
2–5	205	162 (56)	43 (47.3)		178 (57.6)	26 (38.4)	
>5	121	81 (29)	40 (43.9)		82 (27.6)	40 (53.4)	

^*^ p < 0.05.

Biochemistry data of 377 patients with HCC according to mRNA expression were presented in Table 3 and Table 4. ISX and BRD4 were significant in liver capsule and lymphovascular invasion, respectively.

**TABLE 3:** Biochemistry data of 377 HCC cancer patients according to mRNA expression of ISX and BRD4

	Total	ISX			BRD4		
		Low	High	*p*	Low	High	*p*
Type	N = 377						
NBNC	60	46 (16.1)	14 (15.2)	0.675	49 (16.3)	11 (14.9)	0.132
HBV	146	106 (37.2)	40 (43.5)		109 (35.9)	37 (50.0)	
HCV	157	123 (43.2)	34 (37)		134 (44.2)	23 (31.1)	
HBV+HCV	14	10 (3.5)	4 (4.3)		11 (3.6)	3 (4.0)	
AFP	N = 368						
Low	212	162 (58.9)	50 (53.8)	0.3854	173 (59)	39 (52)	0.2707
High	156	113 (41.1)	43 (46.2)		120 (41)	36 (48)	
Bilirubin	N = 226						
≦1.2	184	136 (80.9)	48 (82.8)	0.7604	150 (82)	34 (79)	0.6603
>1.2	42	32 (19.1)	10 (17.2)		33 (18)	9 (21)	
ALB	N = 230						
≦4.5	199	148 (85.6)	51 (89.5)	0.4517	161 (85.6)	38 (90.5)	0.4065
>4.5	31	25 (14.4)	6 (10.5)		27 (14.4)	4 (9.5)	
GOT	N = 227						
<40	100	70 (41.4)	30 (51.7)	0.3674	78 (42.4)	22 (51.2)	0.2069
40–100	90	71 (42)	19 (32.8)		78 (42.4)	12 (27.9)	
>100	37	28 (16.6)	9 (15.5)		28 (15.2)	9 (20.9)	
GPT	N = 229						
<40	103	74 (43.3)	29 (50)	0.5987	80 (43)	23 (53.5)	0.4095
40–100	82	62 (36.3)	20 (34.5)		70 (37.6)	12 (27.9)	
>100	44	35 (20.4)	9 (15.5)		36 (19.4)	8 (18.6)	
Sugar	N = 145						
<100	38	29 (25.4)	9 (29)	0.5377	34 (27.9)	4 (17.4)	0.4402
100–120	43	32 (28.1)	11 (35.5)		34 (27.9)	9 (39.1)	
>120	64	53 (46.5)	11 (35.5)		54 (44.2)	10 (43.5)	
ALP	N = 82						
<40	1	0 (0)	1 (4.2)	0.2894	0 (0)	1 (4.8)	0.1889
40–100	61	44 (75.9)	17 (70.8)		47 (77)	14 (66.7)	
>100	20	14 (24.1)	6 (25)		14 (23)	6 (28.5)	
Lymphovascular invasion	N = 347						
No	207	161 (61.7)	46 (53.5)	0.2438	175 (63.2)	32 (45.7)	0.0075*
Yes	140	100 (38.3)	40 (46.5)		102 (36.8)	38 (54.3)	
Liver capsule invasion	N = 159						
No	97	77 (65.8)	20 (47.6)	0.0381*	78 (62.4)	19 (55.9)	0.4896
Yes	62	40 (34.2)	22 (52.4)		47 (37.6)	15 (44.1)	

^*^ p < 0.05.

**TABLE 4:** Biochemistry data of 377 HCC cancer patients according to mRNA expression of PCAF

	Total	PCAF		
		Low	High	*p*
Type	N = 377			
NBNC	60	46 (16.1)	14 (15.8)	0.7868
HBV	145	106 (37.2)	39 (42.6)	
HCV	157	122 (42.7)	35 (38.6)	
HBV+HCV	15	12 (4.0)	3 (3.0)	
AFP	N = 368			
Low	212	161 (60.1)	51 (51.0)	0.1171
High	156	107 (39.9)	49 (49.0)	
Bilirubin	N = 226			
≦1.2	184	133 (79.2)	51 (87.9)	0.1390
>1.2	42	35 (20.8)	7 (12.1)	
ALB	N = 230			
≦4.5	199	144 (84.2)	55 (93.2)	0.0806
>4.5	31	27 (15.8)	31 (6.8)	
GOT	N = 227			
<40	100	69 (41.0)	31 (52.5)	0.3110
40–100	90	70 (41.7)	20 (33.9)	
>100	37	29 (17.3)	8 (13.6)	
GPT	N = 229			
<40	103	77 (45.3)	26 (44.1)	0.5517
40–100	82	58 (34.1)	24 (40.7)	
>100	44	35 (20.6)	9 (15.2)	
Sugar	N=			
<100	38	28 (25.9)	10 (27.0)	0.9207
100–120	43	33 (30.6)	10 (27.0)	
>120	64	47 (43.5)	17 (46.0)	
ALP				
<40	1	0 (0)	1 (4.8)	0.1972
40–100	61	45 (73.8)	16 (76.2)	
>100	20	16 (26.2)	4 (19.0)	
Lymphovascular invasion				
No	207	156 (61.4)	51 (54.8)	0.3481
Yes	140	98 (38.6)	42 (45.2)	
Liver capsule invasion				
No	97	73 (60.3)	24 (63.2)	0.7552
Yes	62	48 (39.7)	14 (36.8)	

### 2.2 Linear trends of ISX, BRD4, and PCAF show that ISX and BRD4 have a high correlation with the outcome after HCC resection

**FIGURE 1:**
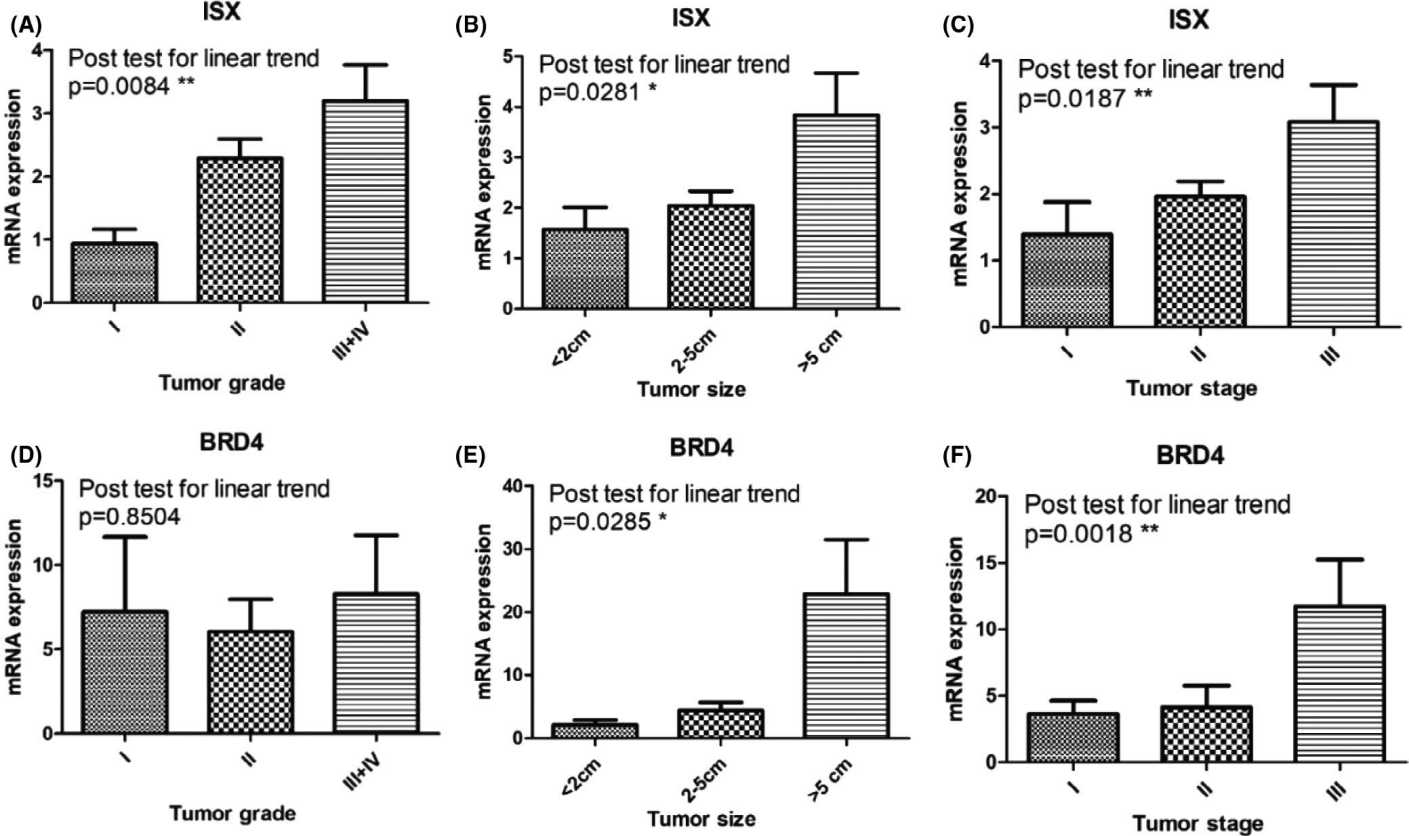
Linear trend analyze of ISX and BRD4. (A‐C), The mRNA expression of ISX has positive correlation with the tumor grade(a), size(B), and stage(C). (D‐F), The mRNA expression of BRD4 has positive correlation with the tumor grade(D), size(E), and stage(F)

To figure out the trend of the significant difference between the three genes in patients with HCC, we ran tests of the linear trend of ISX, BRD4, and PCAF. We found that the mRNA expression of ISX and BRD4 had a high positive correlation with the stage, grade, and size of HCC (Figure 1A–F). However, the expression of PCAF had little influence on the mentioned outcome (Table 5).

**TABLE 5:** Clinical and pathological characteristics of 377 HCC patients according to mRNA expression of PCAF

	Total	PCAF		
		Low	High	*p*
N=	377	275 (%)	102 (%)	
Stage				
I	207	153 (55.6)	54 (52.9)	0.4638
II	107	80 (29.1)	27 (26.5)	
III	63	42 (15.3)	21 (20.6)	
Grade				
I	49	34 (12.5)	15 (14.3)	0.1101
II	247	184 (66.8)	63 (61.2)	
III	79	57 (20.7)	22 (22.5)	
IV	2	0 (0)	2 (2.0)	
Size (cm)				
<2	51	33 (11.8)	18 (17.6)	0.1320
2–5	202	155 (56.8)	47 (46.1)	
>5	124	87 (31.4)	37 (36.3)	

### 2.3 ISX, PCAF, and BRD4 are co‐expressed in HCC cell

**FIGURE 2:**
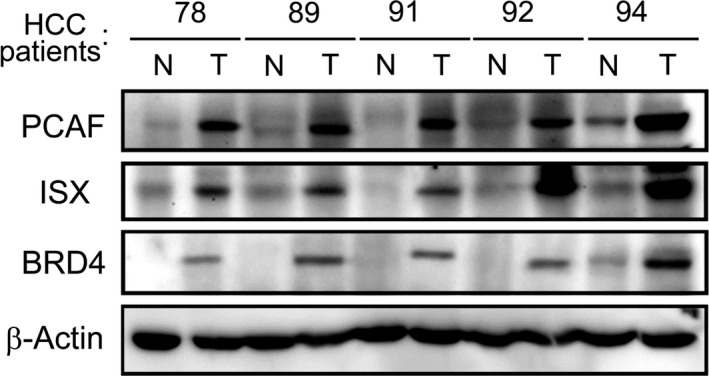
Western blot analysis of ISX, BRD4, and PCAF in HCC tissues (T1‐5) and adjacent liver tissues (A1‐5). Actin was used as a loading control

**FIGURE 3:**
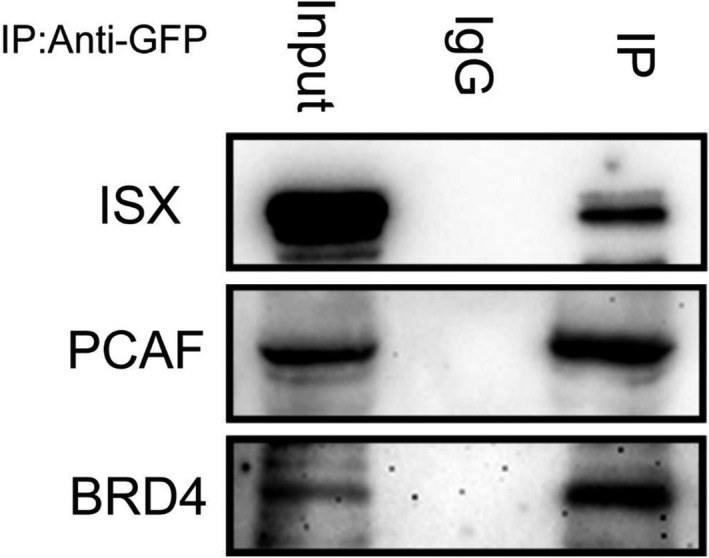
PCAF, BRD4 determined by Western blot in anti‐ISX immunoprecipitates of tumor tissues from patients with liver cancer

**FIGURE 4:**
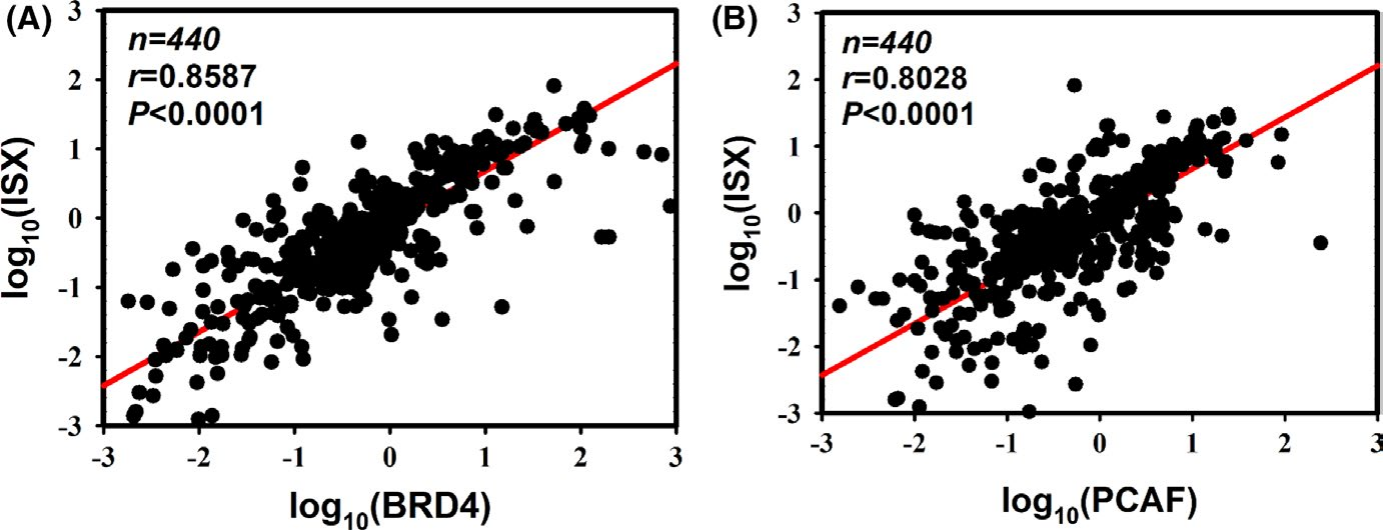
mRNA expression of ISX strongly correlated with those of BRD4(A) and PCAF(B) in patients with HCC (Pearson's correlation coefficient, *r* = 0.8587 and 0.8028, respectively, *p* < 0.0001)

Western blots of four pairs of tumors/adjacent liver tissues confirmed an increased expression of ISX, BRD4, and PCAF in HCC (Figure 2). To analyze the interaction mode of the ISX–BRD4 and ISX–PCAF complexes, co‐immunoprecipitation was used to identify the interaction domain between ISX, BRD4, and PCAF in SK‐Hep1 cells. The result showed that ISX, PCAF, and BRD4 are co‐expressed in HCC cells (Figure 3). Moreover, mRNA expression of ISX strongly correlated with those of BRD4 and PCAF in patients with HCC (Pearson's correlation coefficient, r = 0.8587 and 0.8028, respectively, *p* < 0.0001) (Figure 4A,B).

### 2.4 ISX–BRD4, ISX–PCAF, and BRD4–PCAF analyzed in patients with HCC

In Tables 6, 7, 8, and 9, the clinical and pathological characteristics of 377 patients with HCC according to mRNA expression of ISX–BRD4, ISX–PCAF, PCAF–BRD4, and ISX–PCAF–BRD4 were, respectively, shown. Significant differences in tumor sizes can be observed in Tables 6, 7, 8, and 9; evident differences in cancer stages between patients with high and low levels of ISX or BRD4 expression are also observed in Table 7.

**TABLE 6:** Clinical and pathological characteristics of 377 HCC patients according to mRNA expression of ISX–BRD4

	*Total*	*ISX‐BRD4*				
		Low ISX Low BRD4	High ISX, Low BRD4	High BRD4 Low ISX	High BRD4 High ISX	*p*
N=	377	266(%)	39(%)	16(%)	56(%)	
Stage						
I	207	154 (57.9)	23 (59.0)	8 (50.0)	22 (39.3)	0.0008*
II	107	77 (28.9)	13 (33.3)	4 (25.0)	13 (23.2)	
III	63	35 (13.2)	3 (7.7)	4 (25.0)	21 (37.5)	
Grade						
I	49	37 (14.0)	3 (8.3)	3 (21.4)	6 (8.9)	0.1307
II	247	178 (66.9)	21 (52.8)	12 (71.4)	36 (64.2)	
III	79	50 (18.7)	14 (36.1)	1 (7.2)	14 (26.8)	
IV	2	1(0.04)	1 (2.8)	0 (0)	0 (0)	
Size (cm)						
<2	51	41 (15.6)	3 (7.7)	1 (6.3)	6 (8.8)	0.0061*
2–5	205	153 (57.4)	22 (56.4)	6 (37.5)	24 (40.3)	
>5	121	72 (27.0)	14 (35.9)	9 (56.2)	26 (50.9)	

^*^ p < 0.05.

**TABLE 7:** Clinical and pathological characteristics of 377 HCC patients according to mRNA expression of ISX–PCAF

	Total	ISX‐PCAF				*p*
		Low ISX Low PCAF	High ISX, Low PCAF	High PCAF Low ISX	High PCAF High ISX	
N=	377	242(%)	31(%)	42(%)	62(%)	
Stage						
I	207	135 (55.8)	18 (58.1)	28 (66.7)	26 (42.0)	0.0902
II	107	71 (29.3)	8 (25.8)	10 (23.8)	18 (29.0)	
III	63	36 (14.9)	5 (16.1)	4 (9.5)	18 (29.0)	
Grade						
I	49	33 (13.7)	1 (3.3)	8 (20.0)	7 (10.0)	0.0517
II	247	163 (67.4)	21 (66.7)	27 (65.0)	36 (56.7)	
III	79	46 (18.9)	9 (30.0)	6 (12.5)	18 (31.7)	
IV	2	0 (0)	0 (0)	1 (2.5)	1 (1.6)	
Size (cm)						
<2	51	32 (13.0)	1 (3.1)	11 (26.2)	7 (11.3)	0.0062*
2–5	205	139 (56.1)	19 (62.5)	23 (54.8)	25 (40.3)	
>5	121	72 (30.9)	11 (34.4)	8 (19.0)	30 (48.4)	

^*^ p < 0.05.

**TABLE 8:** Clinical and pathological characteristics of 377 HCC patients according to mRNA expression of PCAF–BRD4

	Total	PCAF‐BRD4				*p*
		Low PCAF Low BRD4	High PCAF, Low BRD4	High BRD4 Low PCAF	High BRD4 High PCAF	
N=	377	238(%)	35(%)	65(%)	39(%)	
Stage						
I	207	135 (56.7)	18 (51.4)	41 (63.1)	13 (33.4)	0.0002*
II	107	72 (30.3)	7 (20.0)	18 (27.7)	10 (25.6)	
III	63	31 (13.0)	10 (28.6)	6 (9.2)	16 (41.0)	
Grade						
I	49	28 (12.2)	6 (15.2)	11 (16.4)	4 (10.3)	0.0991
II	243	156(67.0)	25 (72.7)	39 (60.7)	23 (59.0)	
III	79	54 (22.6)	4 (12.1)	9 (19.6)	12 (30.7)	
IV	2	0(0)	0 (0)	2 (3.3)	0(0)	
Size (cm)						
<2	51	31 (13.2)	1 (2.8)	13 (20.0)	5 (12.8)	0.0012*
2–5	205	142 (59.1)	15 (41.7)	34 (52.3)	14 (35.9)	
>5	121	68 (27.7)	19 (55.6)	14 (35.9)	20 (51.3)	

^*^ p < 0.05.

**TABLE 9:** Clinical and pathological characteristics of 377 HCC patients according to mRNA expression of ISX‐BRD4‐PCAF

	Total	ISX‐BRD4‐PCAF				*p*
		Low ISX Low BRD4 Low PCAF	High ISX, Low BRD4 Low PCAF	High BRD4 Low ISX Low PCAF	High PCAF Low ISX Low BRD4	
N=	377	255(%)	13(%)	17(%)	41(%)	
Stage						
I	207	127 (56.4)	8 (61.5)	8 (47.1)	27 (65.8)	
II	107	67 (29.8)	5 (38.5)	4 (23.5)	10 (24.4)	
III	63	31 (13.8)	0 (0)	5 (29.4)	4 (9.8)	
Grade						
I	49	28 (12.9)	0 (0)	4 (26.7)	8 (20.5)	
II	243	147 (67.4)	6 (50.0)	10 (66.7)	25 (64.1)	
III	79	43(19.7)	6 (50.0)	1 (6.6)	5 (12.8)	
IV	2	0 (0)	0 (0)	0 (0)	1 (2.6)	
Size (cm)						
<2	51	32 (14.0)	0 (0)	0 (0)	10 (24.4)	
2–5	205	132 (57.6)	11 (84.6)	6 (35.3)	23 (56.1)	
>5	121	65 (28.4)	2 (15.4)	11 (64.7)	8 (19.5)	

	Total	ISX‐BRD4‐PCAF				*p*
		High ISX High BRD4 Low PCAF	High ISX High PCAF Low BRD4	High BRD4 High PCAF Low ISX	High PCAF High ISX High BRD4	
N=	377	18(%)	24(%)	1(%)	38(%)	
Stage						
I		10 (55.6)	14 (58.3)	1 (100)	12 (31.6)	0.0052*
II		3 (16.7)	8 (33.3)	0 (0)	10 (26.3)	
III		5 (27.7)	2 (8.4)	0 (0)	16 (42.1)	
Grade						
I		1 (5.6)	2 (9.1)	0 (0)	4 (10.5)	0.0774
II		14 (77.8)	12 (54.6)	1(100)	22 (57.9)	
III		3 (16.7)	7 (31.8)	0 (0)	12 (31.6)	
IV		0 (0)	1 (4.5)	0 (0)	0 (0)	
Size (cm)						
<2		1 (5.3)	3 (12.5)	1 (100)	4 (10.5)	0.0004*
2–5		8 (47.4)	11 (45.8)	0 (0)	14 (36.8)	
>5		9 (47.3)	10 (41.7)	0 (0)	20 (52.6)	

^*^ p < 0.05.

### 2.5 Kaplan–Meier survival curve analysis of patients with HCC shows that patients with HCC having relatively lower ISX and BRD4 expression survive longer

**FIGURE 5:**
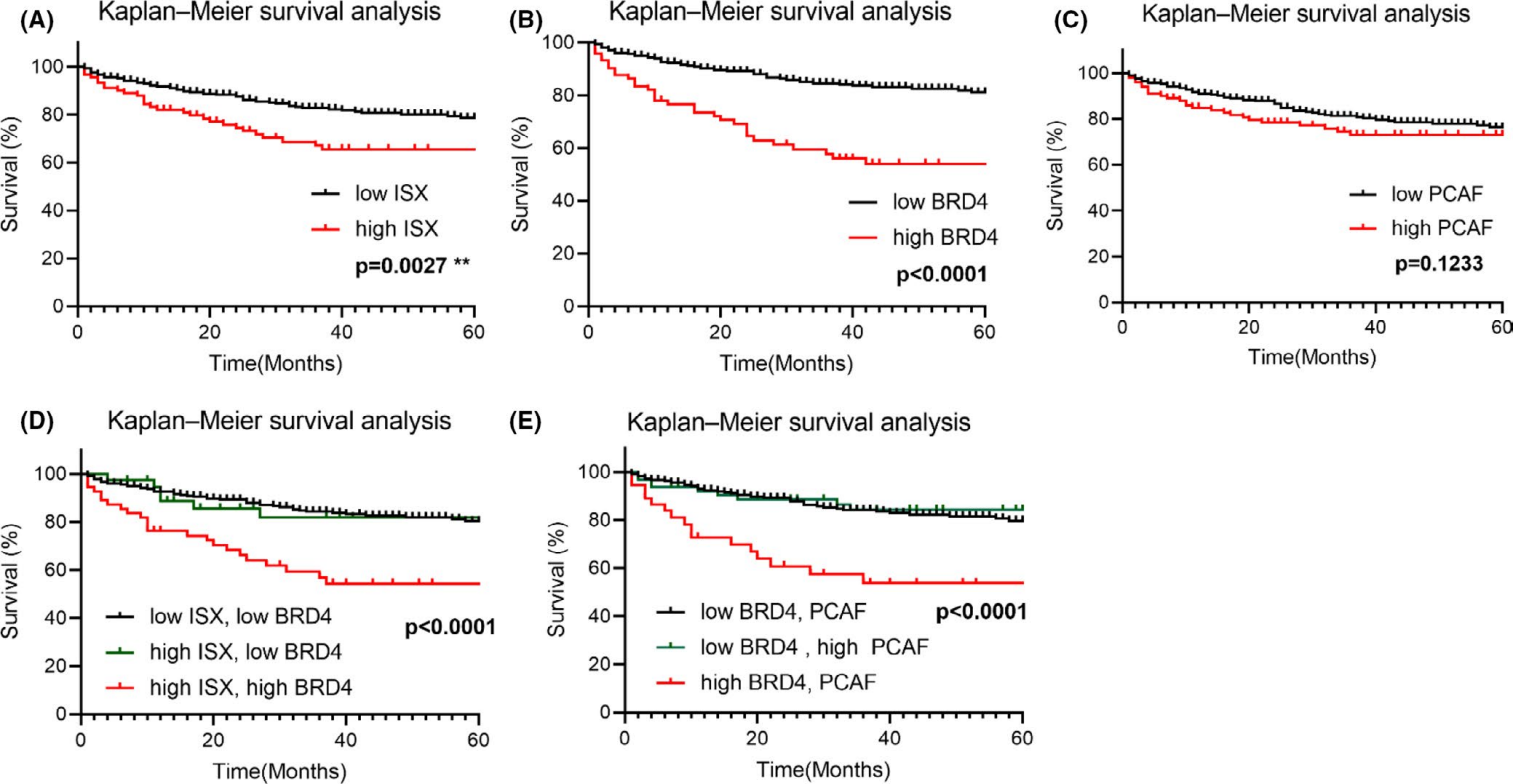
Kaplan–Meier survival curve analysis. (A–C), The Kaplan–Meier survival curve was used to analyze survival correlation between patients (*n* = 377) with HCC and ISX (A),BRD4(B), and PCAF (C) levels. On the basis of the cut‐off values of fold differences, the study population was dichotomized into the “high” and “low” expression groups. *p*‐values were calculated by log‐rank (Mantel–Cox) test comparing the two Kaplan–Meier curves. (D and E), The Kaplan–Meier survival curve was used to analyze survival correlation between patients (n = 377) with HCC and ISX–BRD4 (D) and BRD4–PCAF (E) levels. On the basis of the cut‐off values of fold differences, the study population was dichotomized into the “high” and “low” expression groups. *p*‐values were calculated by log‐rank (Mantel–Cox) test comparing the two Kaplan–Meier curves

An analysis of the survival curves indicated that patients with HCC having relatively lower ISX expression had a significantly higher survival time than that of patients with HCC having relatively higher expression after liver resection (*p *= 0.0027)(Figure 5A). Similarly, patients with HCC having relatively lower BRD4 expression had a significantly longer survival time than patients with HCC having relatively higher expression after liver resection (*p *< 0.0001)(Figure 5B). Nevertheless, the expression of PCAF seems to have less influence on the survival rate of patients with HCC though the survival curve drops slightly (*p *= 0.1233).

The result indicated that PCAF might not be a significant factor to predict the prognosis of patients with HCC; however, the mechanism between PCAF and ISX or BRD4 in HCC remains unclear (Figure 5C).

### 2.6 Higher BRD4 expression in patients with HCC may worsen the prognosis after liver resection

An analysis of the survival curves of the mRNA expression of ISX–BRD4 and PCAF–BRD4 indicated that patients with HCC having relatively lower BRD4, whether its ISX or PCAF was high or low, had a significantly shorter survival time. The results show that BRD4 was a more important factor than ISX to the survival rate of patients with HCC. In other words, the result indicated that patients with HCC with higher BRD4 expression might have a poorer prognosis than those with lower BRD4 expression (Figure 5D,E).

## 3 DISCUSSION

This study indicated that ISX and BRD4 (especially BRD4) were closely associated with the prognosis of patients with HCC. The hypothesis can be verified from the analysis of the clinical and pathological characteristics of 377 patients according to the mRNA expression and the survival curve, which shows that patients with HCC having relatively lower ISX or BRD4 expression had a significantly longer survival time than patients with HCC having relatively higher expression after liver resection. The following analysis of BRD4–ISX and BRD4–PCAF indicated that BRD4 plays the most important role in predicting survival of patients with HCC.

The imaging of PCAF for the prognosis of liver cancer patients is more controversial. In lung cancer, PCAF is reported to form a PCAF–ISX–BRD4 axis with other two proteins, mediating MT signaling and regulating tumor initiation and metastasis,^22^ and promoting cell migration and invasion in lung cancer cells.^23^ However, PCAF is reported in another study to be an anti‐oncogene that plays an important role in the development of HCC by suppressing HCC cell metastasis and EMT by targeting Gli1.^24^ Also, there is a study suggesting that PCAF induces cell death by autophagy.^25^ Although the mRNA expression of ISX strongly correlates with those of BRD4 and PCAF in patients with HCC, denoting that they were co‐expressed in liver cancer cells, the interaction between ISX, BRD4, and PCAF remains to be at issue. In other words, the EMT signaling mechanism regulated by the PCAF–ISX–BRD4 axis, which can be seen in lung cancer seems not to be observed in HCC, or the mechanism is so complicated that it needs further research to elucidate.

It is well known that ISX was already found to have an immunosuppression effect in HCC. In this article, based on the analysis of 377 patients with HCC, BRD4 is proven to be a more important factor than ISX in determining the prognosis of patients after HCC resection. The survival curve clearly separated according to the expression of BRD4 and the linear trend analysis shown that the higher the expression of BRD4, the higher the stage, and larger the tumors. The ISX–BRD4 complex is also a good target for predicting cancer cell metastasis as well as the tumor size.

Collectively, we have shown that ISX, BRD4, and PCAF are co‐expressed in liver cells, and the expression of the ISX–BRD4 complex plays a significant role in determining the prognosis of patients with HCC. The lower the expression of the complex, the higher the survival rate. The advantage of this article is that a large amount of patient data to support the results exists, making them more credible. Our findings highlight the potential of identifying gene expression as a therapeutic target for the prevention of metastasis and improve survival rate.

## 4 MATERIALS AND METHODS

### 4.1 Patients

In this retrospective analysis, we included 377 patients (288 men and 89 women; mean age, 61.2 ± 11.3 years; range, 9–87 years) with confirmed HCC who underwent curative hepatectomy between July 2013 and August 2020 at two medical centers. None of the patients underwent any preoperative treatment. Written informed consent was obtained from each patient. The pathological diagnosis and classification of variables were based on the criteria recommended in the General Rules for Clinical and Pathological Study of Primary Liver Cancer. Clinicopathological characteristics collected for analyses included sex, age, glutamic oxaloacetic transaminase, glutamic‐pyruvic transaminase, albumin, α‐fetoprotein, Bilit, BCLC, tumor stage, tumor size, and tumor number. Tissue specimens obtained during the operation were immediately stored in liquid nitrogen until further analysis. All patients underwent routine and regular follow‐up care at our outpatient department and were carefully monitored once every 6 months for 5 years.

## 5 INCLUSION AND EXCLUSION CRITERIA

The inclusion criterions for the retrospective study were as follows: (a) patients with HCC; (b) no presence of an extrahepatic metastasis; and (c) no presence of any other complications. Among the recruited cases, we excluded patients who received or underwent the following treatments or conditions: (a) treatment(s) with microwave ablation or radiofrequency ablation during surgery; (b) early surgical death within one month; and (c) patients lost to follow‐up. Finally, 377 patients were included in our study.

### 5.1 Quantitative RT polymerase chain reaction

The expression of ISX, BRD4, and PCAF mRNA in HCC cells and cells from cancer patients was quantified using an SYBR Green Quantitative RT–PCR kit (Invitrogen) as described previously. Total RNA was extracted from tumor mass using TRIzol reagent (Invitrogen) and then transcribed into cDNA (Invitrogen) for PCR amplification using a 7900HT Thermocycler (Thermo Fisher Scientific). All procedures and data analyses were performed according to the manufacturers’ instructions. All data are expressed as mean ±SD of at least three experiments.^22^

### 5.2 Cell culture

The human liver cancer cell line, SK‐Hep1, was purchased from the ATCC in June 2020. Cell lines from ATCC have been thoroughly tested and authenticated; morphology, karyotyping, and PCR‐based approaches were used to confirm the identity of the original cell lines. Cells were grown in 90% Eagle Minimum Essential Medium (MEM;Gibco) with 2 mmol/L L‐glutamine and Earle's Balanced Salt Solution (BSS; Gibco) adjusted to contain 1.5 g/L sodium bicarbonate, 0.1 mmol/L nonessential amino acids (Gibco),1.0 mmol/L sodium pyruvate, and 10% FBS (Gibco). All cell lines have been routinely tested for mycoplasma contamination using a Universal Mycoplasma Detection Kit (Thermo Fisher Scientific), and the last mycoplasma test was performed in August 2020. Mycoplasma‐free cell lines were used in all experiments.

### 5.3 Statistical analysis

Patients with HCC were classified into two groups—“low” and “high” according to survival receiver operator characteristic (ROC) curve analysis. The cutting points of ISX, BRD4, and PCAF separately were 2.0, 3.0, and 2.1 times of the mRNA expression in liver cancer tumors than that of the neighboring healthy tissues. Statistical analysis of categorical variables was carried out by one‐way ANOVA. The cut‐off value of ISX, PCAF, and BRD4 was based on the results of previous studies.^22^

Quantitative variables are presented as the mean ± SD. Significant differences were determined using a two‐sample *t*‐test. Pearson's correlational analysis was used to examine the relationship between the levels of ISX, BRD4, and PCAF expression. Statistical analysis of categorical variables was performed using the chi‐square test, one‐way ANOVA, and Fisher's exact test. The Kaplan–Meier survival curve was used to analyze survival correlation between patients with HCC and ISX, BRD4, PCAF ISX–BRD4, and BRD4–PCAF levels. *p*‐values were calculated by log‐rank (Mantel–Cox) test comparing the two Kaplan–Meier curves. *p*‐values < 0.05 were considered statistically significant. Data analysis was performed using JMP software (version 14.0).

### 5.4 Western blotting and immunohistochemical analysis

Western blotting staining and immunohistochemical (fluorescence) staining were performed as described previously.^26^ The primary antibodies used in this study were PCAF (1:1,000 dilution; #3378S; Cell Signaling Technology), b‐actin (1:10,000 dilution; #4967L; Cell Signaling Technology), GFP (1:500 dilution; SC‐9996; Santa Cruz Biotechnology), ISX (1:200 dilution; sc‐398934; Santa Cruz Biotechnology), BRD4 (1:1,000 dilution; #13440S; Cell Signaling Technology). FITC‐conjugated antirabbit IgG, rhodamine‐conjugated anti‐mouse IgG, and alkaline phosphatase‐conjugated anti‐rabbit IgG antibody (1:500 dilution; Jackson ImmunoResearch Laboratories, West Grove, PA, USA) were also used. All experiments were repeated at least three times.

### 5.5 Co‐immunoprecipitation (CO‐IP)

Whole‐cell lysates from 5 × 10^7^ cells were prepared in modified RIPA buffer (50 mM Tris‐Cl (pH7.5), 150 mM NaCl, 1 mM EDTA, 1% NP‐40 (0.1% SDS), and 0.5% Na‐deoxycholate). After centrifugation, the supernatant was incubated with 5 μg of antibodies as indicated, and then, protein A/G Sepharose bead was added, and the incubation was continued at 4°C. The beads were washed five times with 1,000 ml of RIPA buffer and examined by western blot analysis.^22^

## CONFLICT OF INTEREST

There is none

## AUTHOR CONTRIBUTIONS

All listed authors met the ICMJE criteria. All the authors contributed significantly to the creation of this manuscript, each having fulfilled criteria as established by the ICMJE.

## ETHICAL APPROVAL

The study was conducted with approval (KMUH‐IRB‐20130052) from the ethics committee of the Kaohsiung Medical University Chung Ho Memorial Hospital.

## Data Availability

The data that support the findings of this study are available from the corresponding author upon reasonable request.
